# Analysis of the Comprehensive Tensile Relationship in Electrospun Silk Fibroin/Polycaprolactone Nanofiber Membranes

**DOI:** 10.3390/membranes7040067

**Published:** 2017-12-07

**Authors:** Yunlei Yin, Dandan Pu, Jie Xiong

**Affiliations:** 1 School of Materials and Textiles, Zhejiang Sci-Tech University, Hangzhou 310018, China; yinyunlei66@126.com; 2 College of Textile, Henan University of Engineering, Zhengzhou 450007, China; ddpu0301@163.com; 3 Key Laboratory of Advanced Textile Materials and Manufacturing Technology of Ministry of Education, Zhejiang Sci-Tech University, Hangzhou 310018, China

**Keywords:** electrospinning, nanofiber, geometry structure, constitutive relation, mechanical properties

## Abstract

The mechanical properties of electrospun nanofiber membranes are critical for their applications. A clear understanding of the mechanical properties that result from the characteristics of the individual fiber and membrane microstructure is vital in the design of fiber composites. In this reported study, silk fibroin (SF)/polycaprolactone (PCL) composite nanofiber membranes were preparedusing an electrostatic spinning technology. The nanofiber orientation distribution (FOD) of the membrane was analyzed using multi-layer image fusion technology, and the results indicated the presence of an approximately uniform distribution of fibers in the electrospun membranes. The relationship between the single nanofiber and the membrane was established by analyzing the geometrical structure of the cell by employing a representative volume element (RVE) analysis method. The mechanical properties of the 272 nm diameter SF/PCL composite fibers were then predicted using the developed model.

## 1. Introduction

Nanomaterials have become a popular research topic in recent years and in this vein, electrospinning has been recognized as an effective method to produce continuous fibers with diameters in the range of several nanometers to several microns. In electrospinning, which is a fascinating electrostatic fiber fabricating technique, a charged jet flow is first produced in the polymers by high-voltage static electricity, and the charged jet flow is thinly stretched in the static electric field. The associated solvent is evaporated during this process, which solidifies the jet flow finally resulting in the formation of a nanofiber network on the nanofiber collector. Electrospinning facilitates the optimization of the size, shape and orientation of the fibers through the variation of the spinning parameters. Adjusting these variables produces electrospun fiber membranes suitable for the biomedical fields (including artificial organs [1,2], tissue engineering [3], drug delivery systems [4,5] etc.). 

Silk fibroin (SF) exhibits good mechanical strength and has abundant basic amino acids, which has facilitated its wide use in tissue regeneration engineering as a bioactive, dispersed phase in complex biological systems. However, while the tensile strength of SF is very high its elongation at break is low, so applications of SF have been limited [6,7]. Polycaprolactone (PCL) has high mechanical strength, good flexibility, good biological safety and desirable biodegradability, which have also made it acceptable for human medical applications [8]. Blended spinning of SF and PCL has been conducted to capture the synergistic effects of this polymer blend for the preparation of SF/PCL composite fiber membranes. These membranes have exhibited significant cell adhesion and proliferation when used as tissue engineering scaffolds. Moreover, they can also provide sufficient mechanical strength and toughness to assist in cell adhesion, growth, migration and deposition of an extracellular matrix [9,10]. 

It is well known that fiber orientation distribution (FOD) is an important factor in providing geometric structure anisotropy and mechanical anisotropy in fiber membranes [11]. Since the 1990s, some computer image analysis technologies such as boundary transformation [12], hough transform [13], and tomography [14] have been successfully applied to the measurement of the FOD. However, all of these techniques have some associated problems such as, the measured values are clear and distinct only at the fiber boundaries, the measured values are relatively accurate only when the fiber network has few layers, and the cost of measurement is relatively high [15]. Multi-layer image fusion technology can acquire the microscopic images of materials by using a microscope image testing system that includes automatic focusing [16]. This system can collect a series of images in different layers that cover the depth of the target along with automatic focusing, and then conduct fiber edge image processing and refining on the collected image information of the materials. Furthermore, it can automatically calculate the orientation of the fiber networks within 1–180 degree based on a curvilinear integral algorithm. Finally, the original data of orientation distribution and the distribution curve can be obtained. Related research has shown that the image fusion technology can accurately measure the FOD of fiber networks [17].

In order to fully exploit and utilize the potential of nanofibers, their stress-strain behavior experienced during processing and end use must be determined at various structural levels including single nanofiber and multi-nanofiber assemblies. It is very difficult to test the mechanical properties of nanofiber, because the nanometer diameter of electrospun single fiber makes it too fragile. Conducting a direct test of a single fiber is complicated by sample preparation and the requirement of highly sophisticated test devices. Using conventional experimental conditions, high instrument accuracy is required, so that external disturbances will exacerbate test results [18,19,20]. Consequently, very limited studies of the mechanical properties of single nanofibers have been reported in the literature. Hwang et al. [21] designed a hook model by combining a microcantilever using the probes of an atomic force microscope (AFM) and a scanning electron microscope (SEM) to determine the mechanical behavior of electrospun nanofibers. The results showed that the fiber modulus was inversely related to the fiber diameter i.e., the fibers with a small diameter possessed a higher modulus. In related work, Lin et al. [22] designed a new airflow-assisted method to measure the mechanical properties of electrospun fibers. These authors used a synchronized stretching method to test the mechanical properties of electrospun fibers produced using electrospinning. The tension of the fibers was measured by observing their deflection in a transverse airflow with a known velocity. The accuracy of these results was verified by a traditional tensile test or theoretical estimation. In this way, the authors analyzed the mechanical properties of polyoxyethylene, polyamide, polyimide and many other electrospun nanofibers under tension. In addition, this was the first research in the field that reported the tensile mechanical properties of polyimide nanofibers, which provided an important basis for the study of nanofiber mechanical properties. Kolluru et al. [23] investigated the effect of molecular size and fiber diameter on the mechanical properties of polystyrene electrospun fibers subjected to large tensile deformation. The combination of the macromolecular size and submicron diameter of the fibers defined the ultimate ductility of the fibers as well as the effects of reinforcement and toughening. A more direct measurement of electrospun single fibers was also conducted by Tan et al. [24]. These authors used a commercial nano tensile testing system (Agilent Nano UTM, Santa Clara, CA, USA) to conduct their measurements. The authors only reported results for fibers with a diameter above 1 µm.

In light of the challenges faced in the testing of individual nanofiber, it would be desirable to develop a quantitative relationship between the tensile properties of an individual nanofiber and a nanofiber membrane. Therefore, the tensile properties of random nanofiber membranes, which are easily obtained from macroscopic tensile tests of the membrane, can be used as an indicator of the mechanical properties of individual nanofiber [25,26]. Computer modeling of electrospun nanofiber membranes is a difficult task, because of the randomness of the meso structure of the fiber web and its sophisticated mechanical response. These responses often involve large deformations and rotations, bonding and fiber fracture, fiber slippage and the continuous recombination of fiber network topology. Cox [27] developed the first nonwoven material analysis model. This pioneering research dealt with the mechanical behavior of cellulosic paper. The model calculated the elastic constants of an elastic, long-fiber random network using a small deformation framework. In his fiber structure and deformation process, Cox assumed that the fibers were continuous filaments with their elongation running through the entire structure and the fiber deformation included only axial deformation. Bending deformation and other fiber behaviors were not fully integrated into the model. Subsequently, researchers have proposed several fiber web models, but these models have their limitations [28,29,30,31,32,33]. In view of the complexity of the microstructure of the fiber membrane, a representative volume element (RVE) can be used to analyze the mechanical behavior of fiber membranes. Petterson [34] was the first to apply this method to the study of nonwovens where the fibers were considered to be a continuum that were straight and elastic. The orientation of the fibers conformed to a statistical distribution mode and the bonding points of nonwovens were rigid. The force required to deform the fabric was absorbed by the fibers in each unit cell. Then an affine transformation occurred, which contributed to fiber stress that gathered at the fabric corners and formed an orthogonal stress to any plane. The fiber web model analyzed the post-yield properties of fibers and predicted the weakest link in the fiber cell, which could predict the elasticity and post-yield performance of nonwovens using the characteristics, distribution and orientation of the fibers. However, there are many factors that affect the tensile test results of a randomly oriented fiber nonwoven membrane. A model that only considers fiber orientation is not sufficient for prediction of the tensile strength of an individual fiber in a random nonwoven membrane. First of all, the packing density, or the porosity, of a membrane can significantly alter the test results even if the fiber membrane is composed of identical nanofibers. Second, the test result can vary when the sample dimensions of the nanofiber membrane specimen vary due to a change in the number of fibers in the specimen that participate in resisting deformation under tension. Therefore, a practical analysis is needed that can help predict the mechanical properties, particularly tensile properties of a single nanofiber using known testing parameters.

In this reported study, nanofiber membranes were produced by electrospinning. The morphological structure and FOD of these membranes were characterized using thermal field emission scanning electron microscopy (FE-SEM) and the multi-layer image fusion technology. The geometric configurations of an idealized nanofiber membrane were analyzed with all the fibers randomly distributed and oriented. Based on this analysis, a fiber geometry model was developed that related the tensile strength of the nanofiber membrane to a single nanofiber based on the fiber volume fraction and the dimensions of the test specimen. Using the established model, parametric studies were conducted on the factors that dictated the tensile strength of SF/PCL nanofiber membranes, and estimated tensile strength of a single SF/PCL nanofiber with the diameter of 272 nm. The results of this study may be very useful in the designing and dictating the desired mechanical properties of nanofiber membranes.

## 2. Materials and Methods

### 2.1. Materials

Mulberry silk was purchased from Zheng Qiang Textile Co., Ltd. (Zhejiang, China). Mulberry silk was added to an aqueous solution of 0.5 wt % Na_2_CO_3_, the volume (mL) of degumming solution/mass (g) of mulberry silk was 50. The SF solution was boiled for 30 minutes and this step was conducted twice to remove silk glue. Zhang et al. [35] reportedly used 0.5% Na_2_CO_3_ solution (bath ratio 1:50) and boiled the mulberry silk solution for 30 min and then repeated the process three times to remove sericin. In this latter case the sericin was completely separated from its precursor and the high molecular weight of the SF (~390 KD) was reduced to low molecular weight oligomers of SF (~100 KD). The silk fibroin fiber was mixed with Ajisawa’s agent [n(CaCl_2_):n(C_2_H_5_OH):n(H_2_O) = 1:2:8] at 75 ± 1 °C until all the silk fibers were completely dissolved. After filtration, this solution was placed into a dialysis bag MD 25 (Union Carbide Corporation, Houston, TX, USA) and dialyzed in deionized water for three days with changes of water every 4 hours. Finally, the dialyzed fluid was freeze dried in a lyophilizer (Labconco Corporation, Kansas, MO, USA) for five days which yielded a spongy, porous regenerated fibroin protein.

PCL with a viscosity-averaged molecular weight of 80,000 g·mol^−1^ was purchased from Guanghua Weiye Co., Ltd. (Shenzhen, China). A 6 wt % solution of the two SF and PCL polymers (mass ratio 4:1) were dissolved in hexafluoroisopropanol (99.5% purity, Yancheng Dongyang Biological Products Co., Ltd., Jiangsu, China). The solution was stirred for 48 h on a magnetic stirrer (IKA®CM—MAG HS 7, Guangzhou Yike Laboratory Technology Co., Ltd., Guangzhou, China) to produce the spinning solution. The laboratory electrostatic spinning equipment was comprised of a high-voltage power supply (FC60P2, Glassman Company, Houston, TX, USA) and an injection pump (KDS220, KD Scientific Co., Ltd., Holliston, MA, USA). The electrostatic spinning parameters were as follows: voltage 15 KV, spinning flow rate 1.2 mL·h^−1^, receiving distance 12 cm, environment temperature 25 ± 2 °C, and relative humidity 35 ± 5%. The fiber membranes were collected on an electrically grounded sheet of silver paper. Several single fibers produced under the same conditions were collected on paper templates.

### 2.2. Test Methods

The morphology of SF/PCL composite nanofiber membranes was examined using a Vltra55 (Carl Zeiss SMT Pte Ltd., Kembangan, Singapore) FE-SEM. Image-Pro Plus image analysis software was used to measure the diameter of nanofiber with a sampling number of 100. DHU–11 nanofiber orientation image analysis system (Shanghai Beiang Scientific instruments Co., Ltd., Shanghai, China, Donghua University, Shanghai, China) was used to analyze the FOD. 

SF/PCL single nanofiber tensile specimens were prepared by referring to the methods described in the literature [36]. SF/PCL nanofibers were electrospun with strands of the fibers deposited across a rectangular cardboard frame. The frame contained a single nanofiber mounted on the nano mechanical stretching system (Agilent UTM T150, Santa Clara, CA, USA). The cardboard frame was then cut along the discontinuous lines at the two sides of the frame before the test was conducted. 

To conduct the deformed morphological characterization of the fibers using FE-SEM, electrospun fiber membrane samples were mounted on the uniaxial tensile stage with a gauge length of 40 mm in a KES-G1 multifunctional tensile test apparatus (Kato-Tech Company, Osaka, Japan). The tensile strength and the material strain vs tensile strength of membranes were determined according to the ISO 527-1 [37] and ISO 527-3 [38] standard test methods. The membrane was cut into rectangular specimens 50 mm long and 5 mm wide using a razor guided by a straight edge. The thickness of each specimen was determined from the average of five measurements using a film thickness gauge (Shanghai Sixling instrument Factory, Shanghai, China). The thickness of samples ranged from 0.100 to 0.120 mm depending on the region of the membrane from which the sample was got. The specimens were stretched at a constant engineering strain rate of 0.01 s^−1^.

The membrane porosity was determined from the ratio of the measured mass of the specimen to the mass of a fully dense specimen of the same size by measuring the thickness (with constant force), width, and length of the specimen. It has been reported that this method provides results that are similar to the mercury porosimeter method as detailed by Rutledge et al. [39].

$$P=\frac{M_{1}-M_{2}}{M_{1}}\;M_{1}=\left(L\times W\times T\right)\times \rho$$

*P* is the porosity; M_1_ is the mass of a fully compacted specimen of the same size as the M_2_; M_2_ is the measurement of sample quality; ρ is the density of nanofiber membranes.

## 3. Results and Discussion

### 3.1. Morphology and Structural Features of the SF/PCL Nanofiber Membranes

**Figure 1:**
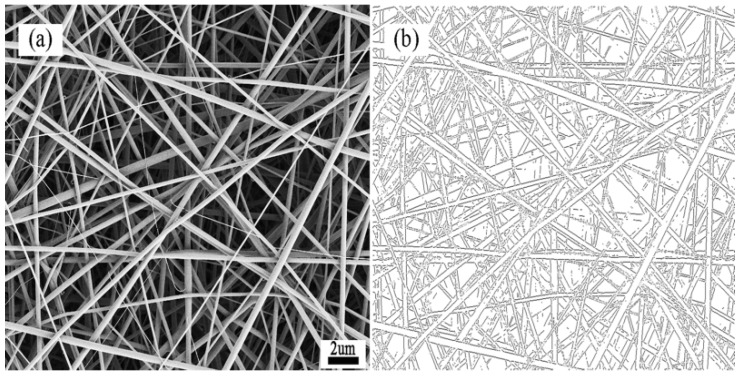
(**a**) SEM of membranes; (**b**) The contours of nanofiber membranes.

**Figure 2:**
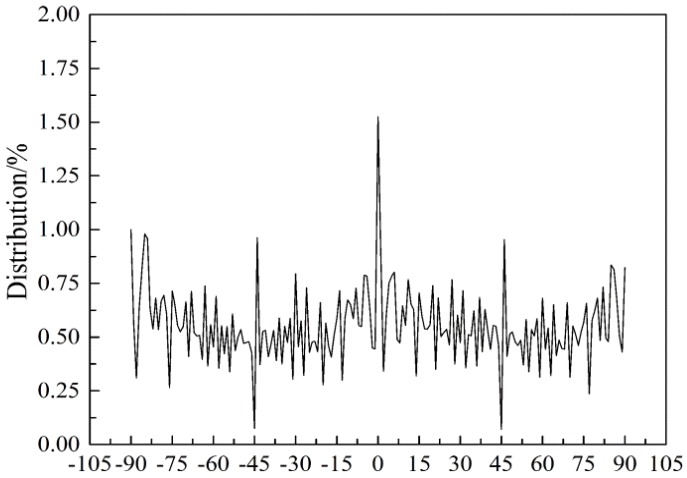
Orientation distribution of silk fibroin/polycaprolactone nanofibers.

In Figure 1a, the SF/PCL nanofibers were present as straight rods, the fiber surface was smooth without beads and the diameter was uniform. As shown, there was no bonding between the fibers at their intersections and the fibers were aligned. In Figure 1b, the contours of nanofiber membranes were observed after processed by the multi-layer fusion technique. It can be seen that the contours of fibers were clear in different depth of the material. Then, according to the integral of the boundary curve, the orientation angle of nanofiber was calculated, and the result of FOD was got by integrating 20 FE-SEM pictures. Based on the Image-Pro Plus images, the diameter of the fibers was found to be mainly in the range of 200–300 nm with an average value of 272 nm and coefficient of variation was 29.4%. To understand the distribution and alignment of the fibers in the fiber membranes, the orientation distribution of the nanofibers was analyzed using the DHU-11 nanofiber orientation image analysis system. The results of the analysis showed that the orientation of fibers in nanofiber membranes approximated an even distribution (Figure 2).

**Figure 3:**
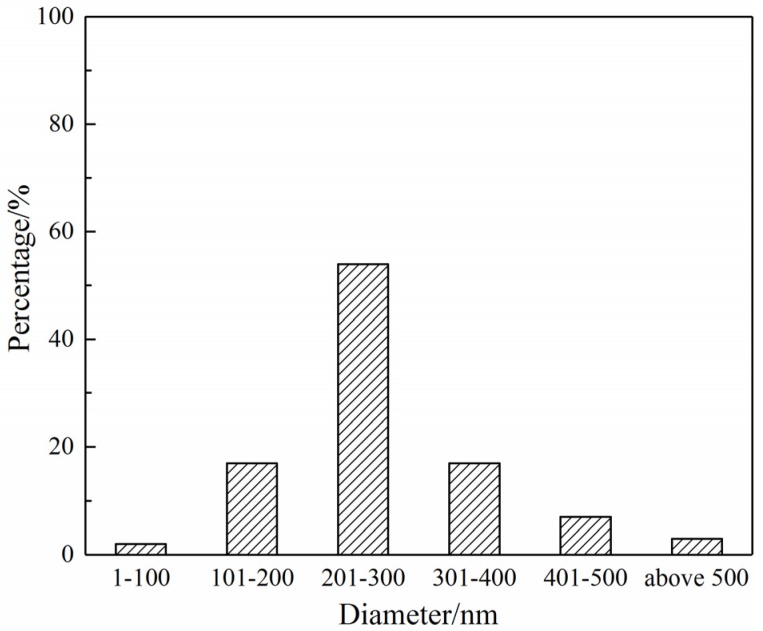
Diameter distribution of nanofibers.

Only fibers with a diameter greater than 1 µm can be tested accurately, because testing of a single nanofiber requires highly precise instruments. However, the nanofiber membranes prepared using HFIP as the spinning solvent often exhibited fiber diameters of about 300 nm (Figure 3), which made it very difficult to directly measure the mechanical properties of SF/PCL single nanofiber. As is well known, when the diameter of a single fiber is reduced to the nanometer level, the scale effect of the fibers becomes apparent. So it is necessary to establish a mesoscale comprehensive mechanical relationship in the nanofiber membranes to predict the mechanical properties of the single nanofiber.

### 3.2. Geometry and Tensile Strength Analysis

**Figure 4:**
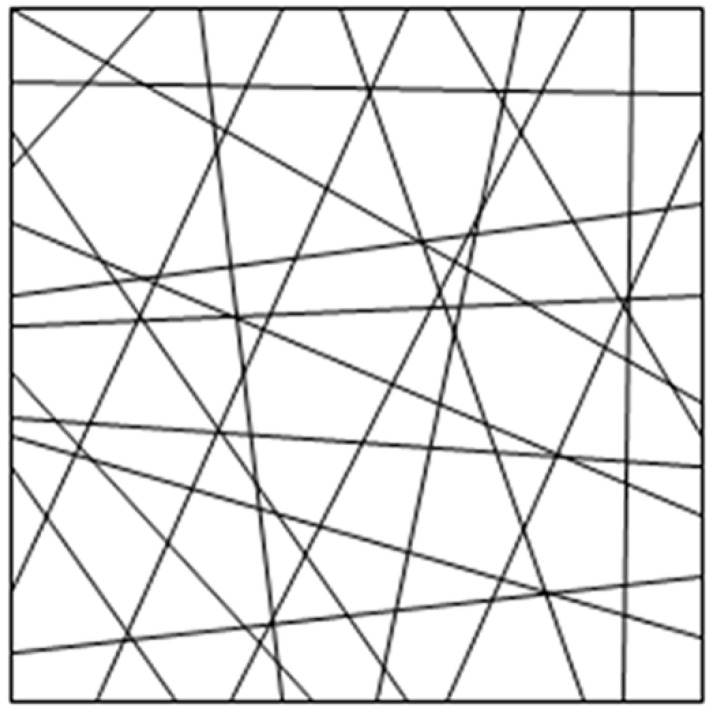
Representative volume element of membranes with nanofibers oriented and distributed.

Selection of the average square RVE represented the complete microstructure of the materials (Figure 4) and could be considered to be a continuous medium, because the geometry of electrospun nanofiber membranes was similar to the traditional nonwovens and the fiber mats were composed of intensive fiber networks.

The structure of SF/PCL nanofiber membranes was theoretically analyzed according to the following assumptions:

1 All the fibers in the RVE had the same diameter and a circular cross section; they were present as straight fibers (the force required for the crimp and buckling of fibers was negligible with respect to the tension of fibers); the materials were incompressible.
2 There was no bonding among fiber intersections in the RVE and the force was negligible; the fiber web was a flat network structure and the fiber orientation was evenly distributed $\psi \left(\alpha \right)=\frac{1}{\pi }$, where *α* was the angle of orientation.
3 The thickness and pore distribution of membranes were uniform and the interaction between the layers could be neglected.
4 The change of fiber length in the RVE was sufficiently regular to be considered to be an arithmetic progression.

#### 3.2.1. Parameter Relationship in RVE

The relationship between the volume of fiber and the volume of fiber membrane can be expressed as:

(1) $$V_{\mu }\left(1-P\right)=V_{f}$$

That is

(2) $$L^{2}T\left(1-P\right)=\pi r^{2}\overset{N_{f}}{\underset{i=1}{\sum }}l_{i}$$

and

(3) $$l_{N_{f}}=\sqrt{2}L$$

(4) $$l_{1}=\Delta l$$

(5) $$\Delta l=\frac{\sqrt{2}L}{N_{f}}$$

(6) $$\overset{N_{f}}{\underset{i=1}{\sum }}l_{i}=N_{f}l_{N_{f}}-\frac{N_{f}\left(N_{f}-1\right)}{2}\Delta l$$

where, *P* is the membrane porosity, $V_{f}$ is the volume of fiber, is the volume of membrane, *L* is the side length of the square, *T* is the membrane thickness, *r* is the fiber radius, $N_{f}$ is the total number of fibers in the RVE, $l_{N_{f}}$ is the length of the *N*th fiber, Δl is tolerances for the fiber length in the RVE. From Equations (2), (3), (5) and (6), we can obtain:

(7) $$N_{f}=\frac{\sqrt{2}LT\left(1-P\right)}{\pi r^{2}}-1$$

#### 3.2.2. Tensile Loading Specimen Analysis

**Figure 5:**
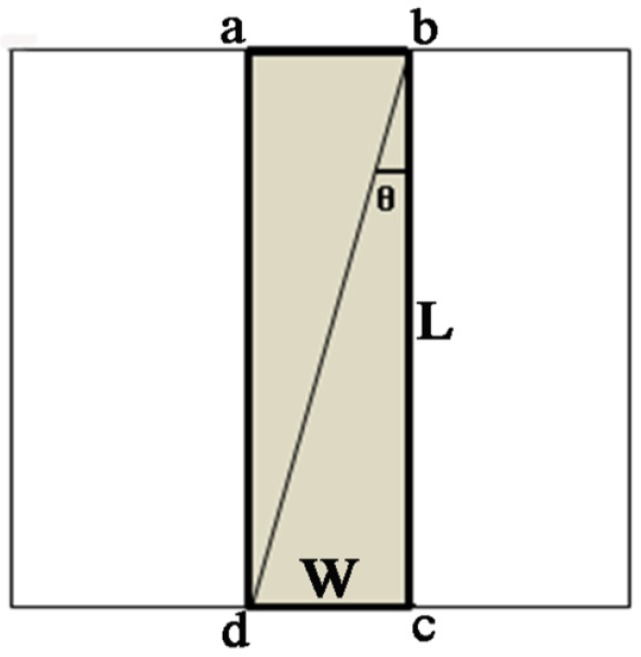
Rectangular tensile testing specimen cut from a RVE.

When a rectangular tensile test sample (shaded area in Figure 5) with a length of L and a width of W is cut from the RVE, only the fibers whose ends coincide or overlap in the cross section of the test sample (line ab and cd) will contribute to the resistance of the tensile loading during the test, because interactions between the nanofibers are neglected. Since each fiber is present in a portion of the circle, the fraction of nanofibers that contributes to the test result will be the real portion of abcd ($A_{l}$, the area in board line as shown in Figure 5) in the RVE. Thus the fraction of nanofibers, τ(l) is:

(8) $$\tau \left(l\right)=\frac{A_{l}}{A_{\mu }}=\frac{L\cdot W}{L^{2}}=\frac{W}{L}$$

$A_{\mu }$ is the area of RVE, $A_{l}$ is area of the sample.

#### 3.2.3. Analysis of Tensile Force of Nanofiber Membranes

**Figure 6:**
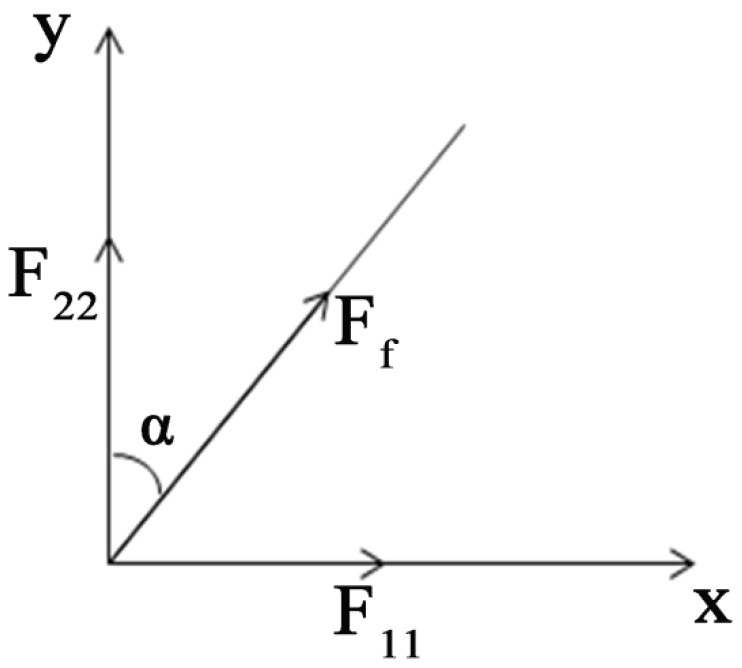
Analysis of single fiber force.

In the uniaxial tensile test, the contribution of a single fiber, which has an orientation angle of α to the direction of stretching of the membrane along the direction of tension. Stress analysis of single fiber is shown in Figure 6:

(9) $$F_{22}=\sigma_{f}\pi r^{2}\cos^{2}\alpha$$

$F_{22}$ is a component provided by a single fiber, which has an angle α to the direction of stretching along the direction of tension; $\sigma_{f}$ is the stress of the single fiber. As shown in Figure 8, by considering the proportion of the tensile specimen in RVE, namely τ(l), the stress *F* in the stretching direction can be obtained from:

(10) $$F=N_{f}\tau \left(l\right)\int_{0}^{\theta }\varphi \left(\alpha \right)F_{22}d\alpha$$

(11) $$F=\frac{\left[\sqrt{2}LT\left(1-P\right)-\pi r^{2}\right]W}{L}\sigma_{f}\int_{0}^{\theta }\varphi \left(\alpha \right)\cos^{2}\alpha d\alpha$$

Because the radius *r* of the single fiber is very small relative to the thickness and length of the specimen, the parameter $\pi r^{2}$ can be ignored. In this case, Equation (11) can be converted into:

(12) $$F=\frac{\sqrt{2}TW\left(1-P\right)\sigma_{f}}{\pi }\int_{0}^{\theta }\cos^{2}\alpha d\alpha$$

Therefore, the stress σ of the membrane can be expressed as:

(13) $$\sigma =\frac{\sqrt{2}\left(1-P\right)\left(\sin\theta \cos\theta +\theta \right)}{2\pi }\sigma_{f}$$

That is

$$\theta =\arctan\frac{W}{L}\;\mathrm{setting}\;k=\frac{W}{L}$$

(14) θ=arctank

(15) $$\sin\theta =\frac{k}{\sqrt{1+k^{2}}}$$

(16) $$\cos\theta =\frac{1}{\sqrt{1+k^{2}}}$$

Equation (13) can be written as:

(17) $$\sigma =\frac{\sqrt{2}\left(1-P\right)\left[k+\left(1+k^{2}\right)\arctan k\right]}{2\pi \left(1+k^{2}\right)}\sigma_{f}$$

Using Equation (17), the stress on the membranes with evenly distributed single fibers was determined primarily by the width-length ratio *k*, the porosity *P* of fiber membranes and the stress on the single fibers. At the same time, it should be noted that the tensile behavior of the fiber membranes was highly dependent on the size of the tensile specimen, and the effect of the porosity of electrospun nanofiber membranes on the uniaxial tensile properties of these membranes must also be considered. If θ was 0, then the stress in the model was 0. This resulted from the corresponding width to length ratio *k*, which was 0 and there no fibers ran through the RVEs.

Based on the reports of Backer and Petterson [31], the strain ε of the fiber web and the strain $\varepsilon_{f}$ of the fiber with an angle of α is formed by:

(18) $$\varepsilon_{f}=\varepsilon \left(\cos^{2}\alpha -v_{xy}\sin^{2}\alpha \right)$$

(19) $$\varepsilon_{f}=\frac{1-v_{xy}k^{2}}{1+k^{2}}\varepsilon$$

where $v_{xy}$ is the contraction coefficient of membrane, and

(20) $$v_{xy}=\frac{\varepsilon_{x}}{\varepsilon_{y}}$$

Therefore, using Equations (17), (19) and (20), the stress-strain relationship between nanofiber membranes and the single nanofiber can be obtained which assisted in the design and establishment of the tensile mechanical model of the membrane.

### 3.3. Predicting the Mechanical Properties of Single Nanofiber

As mentioned previously, the fibers in the SF/PCL nanofiber membranes were distributed approximately evenly, but after the tensile deformation of membrane, the fibers were distributed along the tensile direction and transverse contraction occurred in the fiber membrane. The average diameter of the SF/PCL composite nanofibers was 272 nm. During analysis of membrane tension (Equations (11) and (12)), the parameter $\pi r^{2}$ was very small compared to the membrane thickness *T* and the length *L*, so it was theoretically ignored.

**Figure 7:**
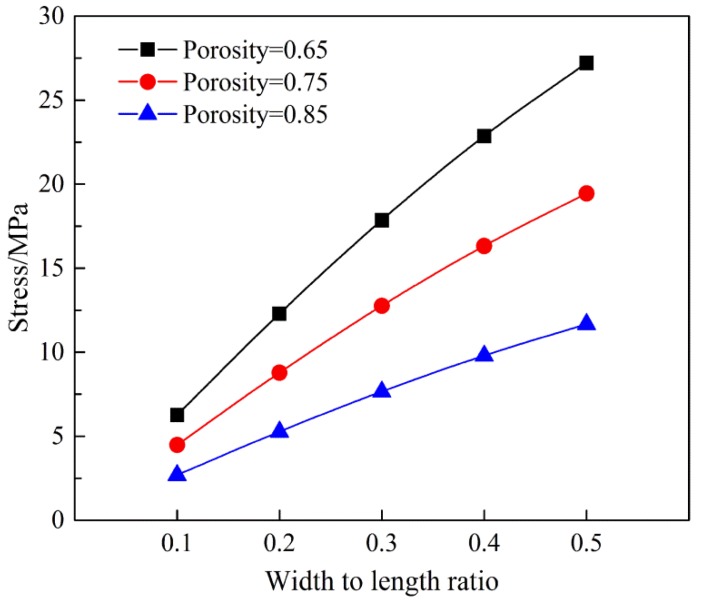
Effects of porosity and width to length ratio on the stress of nanofiber membranes (the stress of a single nanofiber is assumed to be 400 MPa).

At the same time, according to Equation (17), the stress σ was related to porosity *P*, and porosity *P* was associated with the diameter of the nanofibers, but was also related to the thickness *T* of the fiber membranes. It was found that thicker test specimens produced a lower porosity. Figure 7 shows the relationship between the width to length ratio *k*, the porosity *P* and the membrane stress of the specimen. In membranes with a low porosity, the stress was larger, because the increased number of fibers in a unit cell contributed to the force on the membranes.

According to Equation (18), the contraction coefficient $v_{xy}$ of membranes in tensile stress was critical to the analysis of the nanofiber deformation. According to Petterson [34], it is difficult to calculate the deformation during the stretching process of single fibers, because the fibers realign during this process. Since the probability density function of the distribution of fiber orientation was difficult to calculate, it was suitable to express the deformation of fibers by calculating the vertical and horizontal deformation during stretching. 

**Figure 8:**
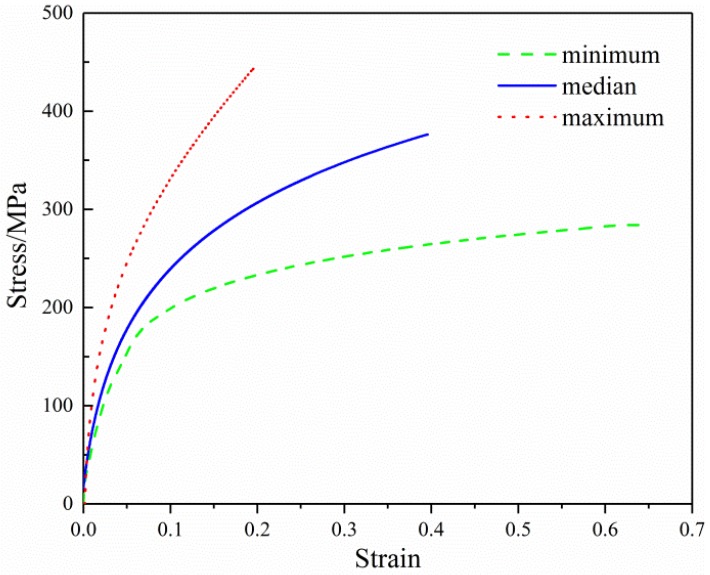
The measured tensile curves of a single nanofiber.

Figure 8 shows the engineering stress-strain curves for single SF/PCL fibers obtained using the Agilent UTM T150 nano tensile tester. More than ten samples were tested, and the median curve represents the calculated weighted average results for the samples. The engineering stress-strain curves were smoothed for a cleaner presentation. It can be seen from this graph that the mechanical properties of the SF/PCL nanofiber were significantly different. It is believed that the main reason for this result was that there were large variations between the external morphology and internal structure of the nanofibers. In this test, ten specimens were tested and the tensile properties of SF/PCL nanofiber membranes were shown in Table 1.

**Figure 9:**
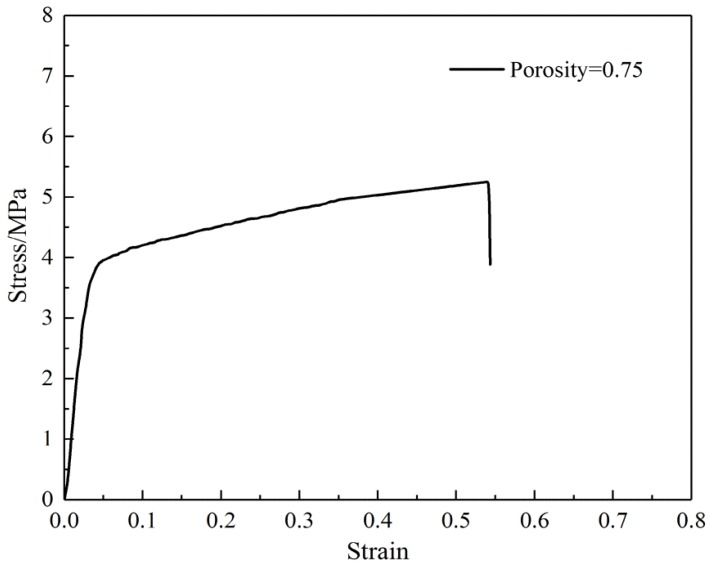
The measured tensile curves of silk fibroin/polycaprolactone nanofiber membranes.

**Figure 10:**
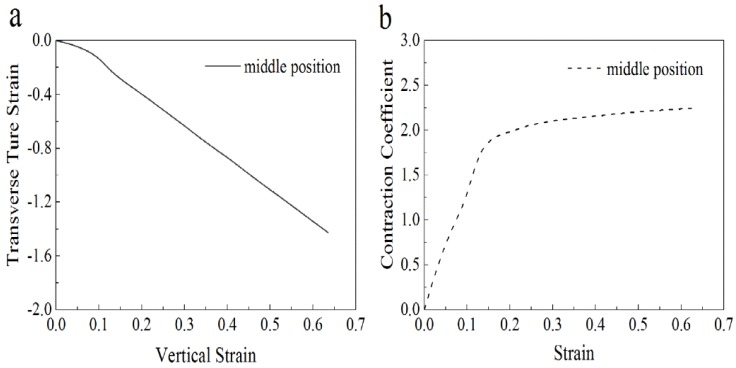
Uniaxial tensile characterization of membranes: (**a**) axial strain versus transverse strain; (**b**) axial strain versus contraction coefficient.

To predict the mechanical properties of the SF/PCL nanofibers, membranes with a width to length ratio of *k* = 0.125 and a porosity of *P* = 0.75 were prepared. A test was performed on these membranes based on ISO 527-3 [38], using standard atmospheric conditions with a tensile rate of 0.01 s^−1^ applied to the test specimens. In this test, five specimens were examined and the measured tensile properties of the SF/PCL nanofiber membranes are shown in Table 2. A representative tensile curve is shown in Figure 9, and the change in the contraction coefficient $v_{xy}$ during stretching is shown in Figure 10.

As can be seen from Table 3, when the yield stress of the membranes was 4.11 MPa, the predicted stress on a single nanofiber reached 295 MPa and the strain of nanofibers was slightly lower than the tested membranes. Based on this analysis, the strength and elongation of single nanofiber predicted by the structure model were slightly higher than the actual tested values. This inequity may have resulted from the theoretical assumptions in the model, where fibers in the RVEs were considered to be straight, without bends or crimps and the force on the fiber intersections was ignored. These conditions may well have resulted in predicted values were higher than measured values.

## 4. Conclusions

To predict the mechanical properties of nanofiber, a geometric comprehensive model was established based on the structure of select membranes. Using this model and the selected RVEs, the meso structure of the membranes was reasonably simplified and analyzed. The analytical results showed that the mechanical properties of nanofibers membranes with a uniform FOD were mainly determined by the size and porosity of the test specimen. A nanofiber membrane with a high width to length ratio and low porosity tends to exhibit high strength.

The model was further used to predict the mechanical properties of an electrospun SF/PCL nanofiber. The results showed that with a fiber diameter of 272 nm, the fracture stress of the SF/PCL nanofiber could attain about 400 MPa and a significant nanoscale effect was exhibited in the tensile properties of a single nanofiber.

## Figures and Tables

**Table 1:** The mechanical properties of silk fibroin/polycaprolactone nanofibers.

Property (Unit)	Average	Minimum	Maximum	CV
Elastic modulus (MPa)	4800	3500	6200	0.23
Yield stress (MPa)	190	170	212	0.08
Yield strain	0.040	0.034	0.048	0.15
Break stress (MPa)	375	285	440	0.17
Break strain	0.39	0.20	0.63	0.40

**Table 2:** Mechanical properties of silk fibroin/polycaprolactone nanofiber membranes.

Property (Unit)	Average	Minimum	Maximum	CV
Elastic modulus (MPa)	98	86	110	0.10
Yield stress (MPa)	4.11	3.88	4.33	0.05
Yield strain	0.049	0.044	0.052	0.07
Post-yield slope (MPa)	2.60	2.29	3.25	0.15
Break stress (MPa)	5.53	5.38	6.02	0.05
Break strain	0.543	0.378	0.725	0.25

**Table 3:** Comparison of mechanical properties of silk fibroin/polycaprolactone nanofibers.

Sample	Elastic Modulus (MPa)	Yield Stress (MPa)	Yield Strain	Break Stress (MPa)	Break Strain
Tested membranes	98	4.11	0.049	5.53	0.543
Tested nanofibers	5500	242.58	0.044	376.36	0.396
Predicted nanofibers	6280	295.24	0.047	397.25	0.516
